# Constraints on tidal charge of the supermassive black hole at the Galactic Center with trajectories of bright stars

**DOI:** 10.1140/epjc/s10052-018-6166-5

**Published:** 2018-08-29

**Authors:** Alexander F. Zakharov

**Affiliations:** 1 National Astronomical Observatories of Chinese Academy of Sciences, 20A Datun Road, Beijing, 100012 China; 2 Institute of Theoretical and Experimental Physics, B. Cheremushkinskaya, 25, Moscow, 117218 Russia; 3 National Research Nuclear University MEPhI (Moscow Engineering Physics Institute), Kashirskoe highway 31, Moscow, 115409 Russia; 4 Joint Institute for Nuclear Research, Dubna, Russia; 5 North Carolina Central University, Durham, NC 27707 USA

## Abstract

As it was pointed out recently in Hees et al. (Phys Rev Lett 118:211101, 2017), observations of stars near the Galactic Center with current and future facilities provide an unique tool to test general relativity (GR) and alternative theories of gravity in a strong gravitational field regime. In particular, the authors showed that the Yukawa gravity could be constrained with Keck and TMT observations. Some time ago, Dadhich et al. (Phys Lett B 487:1, 2001) showed that the Reissner–Nordström metric with a tidal charge is naturally appeared in the framework of Randall–Sundrum model with an extra dimension ($Q^2$ is called tidal charge and it could be negative in such an approach). Astrophysical consequences of presence of black holes with a tidal charge are considerered, in particular, geodesics and shadows in Kerr–Newman braneworld metric are analyzed in Schee and Stuchlík (Intern J Mod Phys D 18:983, 2009), while profiles of emission lines generated by rings orbiting braneworld Kerr black hole are considered in Schee and Stuchlík (Gen Relat Grav 52:1795, 2009). Possible observational signatures of gravitational lensing in a presence of the Reissner–Nordström black hole with a tidal charge at the Galactic Center are discussed in papers (Bin-Nun in Phys Rev D 81:123011, 2010; Bin-Nun in Phys Rev D 82:064009, 2010; Bin-Nun in Class Quant Grav 28:114003, 2011). Here we are following such an approach and we obtain analytical expressions for orbital precession for Reissner–Nordström–de-Sitter solution in post-Newtonian approximation and discuss opportunities to constrain parameters of the metric from observations of bright stars with current and future astrometric observational facilities such as VLT, Keck, GRAVITY, E-ELT and TMT.

## Introduction

The Galactic Center is a very peculiar object. A couple of different models have been suggested for it, including dense cluster of stars [8], fermion ball [9], boson stars [10, 11], neutrino balls [12]. Later, some of these models have been constrained with subsequent observations [8]. However, as it was found in computer simulations, sometimes differences for alternative models may be very tiny as it was shown in paper [13] where the authors discussed shadows for boson star and black hole models. The most natural and generally accepted model for the Galactic Center is a supermassive black hole (see, e.g. recent reviews [14–17]). A natural way to evaluate a gravitational potential is to analyze trajectories of photons or test particles moving in the potential. Shapes of shadows forming by photons moving around black holes were discussed in [18–21] (see also [22]). Shadows (dark spots) can not be detected but theoretical models could describe a distribution of bright structures around these dark shadows. Bright structures around shadows are being observing with an improving accuracy of current and forthcoming VLBI facilities in mm-band, including the Event Horizon Telescope [24–27].

To create an adequate theoretical model for the Galactic Center astronomers monitored trajectories of bright stars (or clouds of hot gas) using the largest telescopes VLT and Keck with adaptive optics facilities [28–34]. One could introduce a distance between observational data for trajectories of bright stars and their theoretical models. Practically, such a distance is a measure of quality for a theoretical fit. To test different theoretical models one of the most simple approach is to compare apocenter (pericenter) shifts for theoretical fits and observational data for trajectories. If an apocenter (pericenter) shifts for a theoretical fit exceed apocenter (pericenter) shifts obtained from observations one should rule out these interval for parameters for theoretical fits. Based on such an approach one could evaluate parameters of black hole, stellar cluster and dark matter cloud around the Galactic Center because if there is an extended mass distribution inside a bright star orbit in addition to black hole, the extended mass distribution causes an apocenter shift in direction which is opposite to relativistic one [35, 36]. One could also check predictions of general relativity or alternative theories of gravity. For instance, one could evaluate constraints on parameters of $R^n$ theory, Yukawa gravity and graviton masses with trajectories of bright stars at the Galactic Center because in the case of alternative theories of gravity a weak gravitational field limit differs from Newtonian one, so trajectories of bright stars differ from elliptical ones and analyzing observational data with theoretical fits obtained in the framework of alternative theories of gravity one constrains parameters of such theories [37–42] (see, also discussion of observational ways to investigate opportunities to find possible deviations from general relativity with observations of bright stars at the Galactic Center [1, 43]).

In paper [2] it was shown that the Reissner–Nordström metric with a tidal charge could arise in Randall–Sundrum model with an extra dimension. Braneworld black holes are considered assuming that they could substitute conventional black holes in astronomy, in particular, geodesics and shadows in Kerr–Newman braneworld metric are analyzed in [3], while profiles of emission lines generated by rings orbiting braneworld Kerr black hole are considered in [4]. Later it was proposed to consider signatures of gravitational lensing assuming a presence of the Reissner–Nordström black hole with a tidal charge at the Galactic Center [5–7]. In paper [44] analytical expressions for shadow radius of Reissner–Nordström black hole have been derived while shadow sizes for Schwarzschild–de Sitter (Köttler) metric have been found in papers [45, 47]. In the present paper for a particle motion in Reissner–Nordström–de-Sitter metric we derive analytical expressions for orbital precession and discuss constraints on tidal charge from current and future observations of bright stars near the Galactic Center.

## Basic notations

We use a system of units where $G = c= 1$. The line element of the spherically symmetric Reissner–Nordström–de-Sitter metric is

1 $$\begin{aligned} d s^2 = - f(r) d t^2 + f(r)^{-1} d r^2 + r^2 d\theta ^2 + r^2 \sin ^2\theta d\phi ^2 ,\nonumber \\ \end{aligned}$$

where function *f*(*r*) is defined as

2 $$\begin{aligned} f(r) = 1 - \frac{2M}{r} + \frac{Q^2}{r^2} - \frac{1}{3} \Lambda r^2. \end{aligned}$$

Here *M* is a black hole mass, *Q* is its charge and $\Lambda$ is cosmological constant. In the case of a tidal charge [2], $Q^2$ could be negative. Similarly to [45, 46, 48, 49], geodesics could be obtained the Lagrangian

3 $$\begin{aligned} \mathcal{{L}}= -\frac{1}{2} g_{\mu \nu } \frac{d x^\mu }{d \lambda } \frac{d x^\nu }{d \lambda }, \end{aligned}$$

where $g_{\mu \nu }$ are the components of metric (1 and $\lambda$ is the affine parameter. There are three constants of motion for geodesics which come from the metric (1), namely

4 $$\begin{aligned} g_{\mu \nu } \dfrac{d x^\mu }{d \lambda } \dfrac{dx^\nu }{d \lambda }=-m, \end{aligned}$$

which is a test particle mass and two constants connected with an independence of the metric on $\phi$ and *t* coordinates, respectively

5 $$\begin{aligned} g_{\phi \nu } \frac{d x^\nu }{d \lambda }=h, \end{aligned}$$

and

6 $$\begin{aligned} g_{t \nu } \frac{d x^\nu }{d \lambda }=E. \end{aligned}$$

For vanishing $\Lambda$-term these integrals of motion (*h* and *E*) could be interpreted as angular momentum and energy of a test particle, respectively. Geodesics for massive particles could be written in the following form

7 $$\begin{aligned} r^4 \left( \dfrac{dr}{d \lambda }\right) ^2 = E^2r^4 - \Delta (m^2r^2+h^2), \end{aligned}$$

where

8 $$\begin{aligned} \Delta =\left( 1-\frac{1}{3}\Lambda r^2\right) r^2 - 2M r+Q^2. \end{aligned}$$

or we could write Eq. (7) in the following form

9 $$\begin{aligned} r^4 \left( \dfrac{dr}{d \tau }\right) ^2= & {} (\hat{E}^2-1)r^4 +{2Mr^3}- Q^2r^2 \nonumber \\&+\frac{1}{3} \Lambda r^6 -\hat{h}^2\left( r^2-\frac{\Lambda }{3}r^4-2Mr+Q^2\right) ,\nonumber \\ \end{aligned}$$

where $\tau =m \lambda$ is the proper time, $\hat{E}=\dfrac{E}{m}$ and $\hat{h}=\dfrac{h}{m}$. We will omit symbol $\wedge$ below.

Since

10 $$\begin{aligned} r^4 \left( \dfrac{d \phi }{d \tau }\right) ^2 = h^2, \end{aligned}$$

one could obtain

11 $$\begin{aligned} \left( \dfrac{d r}{d \phi }\right) ^2= & {} \frac{1}{{h}^2}({E}^2-1)r^4 +\frac{2Mr^3}{{h}^2}- \frac{Q^2r^2}{{h}^2} \nonumber \\&+\frac{1}{3h^2} \Lambda r^6 -\left( r^2-\frac{\Lambda }{3}r^4-2Mr+Q^2\right) , \end{aligned}$$

It is convenient to introduce new variable $u=1/r$. Since

12 $$\begin{aligned} \left( \dfrac{d u}{d \lambda }\right) ^2 = \left( \dfrac{d r}{d \phi }\right) ^2 u^4, \end{aligned}$$

one obtains

13 $$\begin{aligned} \left( \dfrac{d u}{d \lambda }\right) ^2= & {} \frac{1}{h^2}({E}^2-1) +\frac{2Mu}{{h}^2}- \frac{Q^2u^2}{{h}^2} +\frac{\Lambda }{3h^2 u^2} \nonumber \\&-\left( u^2-\frac{\Lambda }{3}-2Mu^3+Q^2u^4\right) , \end{aligned}$$

therefore,

14 $$\begin{aligned} \dfrac{d^2 u}{d \lambda ^2} +u = \frac{M}{{h}^2}+ 3Mu^2 - \frac{Q^2u}{{h}^2} -2Q^2u^3 -\frac{\Lambda }{3h^2 u^3} , \end{aligned}$$

and as it is noted in [50] the first term in the right hand side of Eq. (14) corresponds to the Newtonian case, the second term corresponds to the GR correction from the Schwarzschild metric (see also book [56]), meanwhile one could see inspecting Eq. (14) that third and forth term correspond to a presence of *Q* parameter in metric (1), the fifth term corresponds to a $\Lambda$-term presence in the metric. Assuming that second, third, forth and fifth terms in the right hand side of Eq. (14) are small in respect to the basic Newtonian solution, one could evaluate relativistic precession for each term and after that one has to calculate an algebraic sum of all shifts induced by different terms.

## Relativistic precession evaluation

An impact of non-vanishing charge in Reissner–Nordström metric on orbital precession was discussed in papers [51–55] considering perturbations of Schwarzschild metric, see for instance [56, 57]. However, Eq. (14) was not considered in these papers. When people discussed astrophysical consequences of this effect they evaluated an impact of Solar charge on Mercury precession orbit [51] and it is clear that the effect is very small due to constraints on Solar electric charge. Similarly, for astrophysical black holes including the black holes at the Galactic Center, their electric charges are expecting to be vanishing or very small. However, significant tidal charges |*Q*| which are comparable with *M* are discussed in the literature [6, 7] where the author discussed an opportunity to evaluate a tidal charge $Q^2 \approx - 6.4 M^2$ or $Q^2 \approx 1.6 M^2$ from gravitational lensing.

An expression for apocenter (pericenter) shifts for Newtonian potential plus small perturbing function is given as a solution in the classical (L & L) textbook [58] (see also applications of the expressions for calculations of stellar orbit precessions in presence of the the supermassive black hole and dark matter at the Galactic Center [59, 60]). In paper [50], the authors derived the expression which is equivalent to the (L & L) relation and which can be used for our needs. According to the procedure proposed in [50] one could re-write Eq. (14) in the following form

15 $$\begin{aligned} \dfrac{d^2 u}{d \tau ^2} +u = \frac{M}{{h}^2}- \frac{g(u)}{{h}^2}, \end{aligned}$$

where *g*(*u*) is a perturbing function which is supposed to be small and it could be presented as a conservative force in the following form

16 $$\begin{aligned} g(u)=r^2 F(r)|_{r=1/u}, \quad F(r)=-\frac{dV}{dr}. \end{aligned}$$

For potential $V(r)=\dfrac{\alpha _{-(n+1)}}{r^{-(n+1)}}$ (where *n* is a natural number) one obtains [50]

17 $$\begin{aligned} \Delta \theta (-(n+1))=\frac{-\pi \alpha _{-(n+1)}\chi ^2_n(e)}{ML^n}, \end{aligned}$$

where

18 $$\begin{aligned} \chi ^2_n(e)=n(n+1)_2F_1\left( \frac{1}{2}-\frac{n}{2},\frac{1}{2}-\frac{n}{2},2,e^2\right) , \end{aligned}$$

$_2F_1$ is the Gauss hypergeometrical function, *L* is the semilatus rectum ($L=h^2/M$) and we have $L=a(1-e^2)$ (*a* is semi-major axis and *e* is eccentricity). An alternative approach for evaluation of pericenter advance within of Rezzolla–Zhidenko (RZ) parametrization [61] has been described in [62] for theoretical analysis of pulsar timing in the case if pulsars are moving in the strong gravitational field of the supermassive black hole at the Galactic Center. Since pulsars are very precise and stable clocks, studies of pulsar timing gives an opportunity to investigate gravitational field in the vicinity of the supermassive black hole.

In paper [50] the authors obtained orbital precessions for positive powers of perturbing function

19 $$\begin{aligned} \Delta \theta (n)=\frac{-\pi \alpha _{n}a^{n+1} \sqrt{1-e^2}\chi ^2_n(e)}{M}. \end{aligned}$$

For GR term in Eq. (14) the perturbing potential is $V_{GR}(r)=-\dfrac{Mh^2}{r^3}$ and one obtains the well-known result $n=2$ (see, for instance [50] and textbooks on GR)

20 $$\begin{aligned} \Delta \theta (GR) := \Delta \theta (-(3))=\frac{6\pi M}{L}. \end{aligned}$$

For the third term in Eq. (14) one has potential $V_{RN1}(r)=\dfrac{Q^2}{2r^2}$ ($\alpha _{-2}=\dfrac{Q^2}{2}$ and $n=1$), therefore, one obtains

21 $$\begin{aligned} \Delta \theta (RN1) := \Delta \theta (-(2))_{RN1}=-\frac{\pi Q^2}{ML}. \end{aligned}$$

Equation (21) was derived earlier in [51] to evaluate an impact of Solar charge on orbital precession of Mercury, however, we re-derive the Eq. (21) following a procedure suggested in [58] since this approach is more clear and it could be applied for other types of perturbing potentials. For the forth term in Eq. (14) one has potential $V_{RN2}(r)=\dfrac{h^2Q^2}{2r^4}$ ($\alpha _{-4}=\dfrac{h^2Q^2}{2}$ and $n=3$) , therefore, one obtains

22 $$\begin{aligned} \Delta \theta (RN2) := \Delta \theta (-(4))_{RN2}=-\frac{3\pi Q^2 (4+e^2)}{2L^2}. \end{aligned}$$

Since according to our assumptions $M \ll L$, one has $\dfrac{Q^2}{L^2} \ll \dfrac{Q^2}{ML}$ and we ignore the apocenter (pericenter) shift which is described with Eq. (22). For the fifth (de-Sitter or anti-de-Sitter) term in Eq. (14) one has potential $V_{dS}(r)=-\dfrac{\Lambda r^2}{6}$ ($\alpha _2=-\dfrac{\Lambda }{6}$) and one has the corresponding apocenter (pericenter) shift

23 $$\begin{aligned} \Delta \theta (\Lambda ) := \Delta \theta (2)_{dS}=\frac{\pi \Lambda a^3 \sqrt{1-e^2}}{M}. \end{aligned}$$

Equation (23) was derived earlier in [63] and re-derived in [50] with (L & L) approach [58]. In paper [64] Eq. (23) was used to discuss consequences of a non-vanishing $\Lambda$-term from observations in Solar system.

Therefore, a total shift of a pericenter is

24 $$\begin{aligned} \Delta \theta (total) := \frac{6\pi M}{L} -\frac{\pi Q^2}{ML} + \frac{\pi \Lambda a^3 \sqrt{1-e^2}}{M}. \end{aligned}$$

and one has a relativistic advance for a tidal charge with $Q^2 < 0$ and apocenter shift dependences on eccentricity and semi-major axis are the same for GR and Reissner–Nordström advance but corresponding factors (${6\pi M}$ and $-\dfrac{\pi Q^2}{M}$) are different, therefore, it is very hard to distinguish a presence of a tidal charge and black hole mass evaluation uncertainties. For $Q^2 > 0$, there is an apocenter shift in the opposite direction in respect to GR advance. As it was noted each term in Eq. (24) was known earlier, but people did not consider them together perhaps because of small values electric charge and $\Lambda$-term. However, a wider range for tidal charge was considered for the black hole at the Galactic Center [6, 7] and an excellent precision of astrometrical observations has been reached in last years and it gives an opportunity to evaluate parameters of alternative theories of gravity with these observations.

## Estimates

As it was noted by the astronomers of the Keck group [1], pericenter shift has not be found yet for S2 star, however, an upper confidence limit on a linear drift is constrained

25 $$\begin{aligned} | {\dot{\omega }}| < 1.7 \times 10^{-3} \mathrm{rad}/\mathrm{year}. \end{aligned}$$

at 95% C.L., while GR advance for the pericenter is [43]

26 $$\begin{aligned} | {\dot{\omega }}_{GR}| = \frac{6\pi GM}{Pc^2(1-e^2)}=1.6 \times 10^{-4} \mathrm{rad}/\mathrm{year}, \end{aligned}$$

where *P* is the orbital period for S2 star (in this section we use dimensional constants *G* and *c* instead of geometrical units). Based on such estimates one could constrain alternative theories of gravity following the approach used in [1].

### Estimates of (tidal) charge constraints

Assuming $\Lambda =0$ we consider constraints on $Q^2$ parameter from previous and future observations of S2 star. One could re-write orbital precession in dimensional form

27 $$\begin{aligned} {\dot{\omega }}_{RN} = \frac{\pi Q^2}{PGML}, \end{aligned}$$

where *P* is an orbital period. Taking into account a sign of pericenter shift for a tidal charge with $Q^2 < 0$, one has

28 $$\begin{aligned} {\dot{\omega }}_{RN} < 1.54 \times 10^{-3} \mathrm{rad}/\mathrm{year} \approx 9.625~ {\dot{\omega }}_{GR}, \end{aligned}$$

therefore,

29 $$\begin{aligned} -57.75 M^2< Q^2 <0, \end{aligned}$$

with $95 \%$ C. L. For $Q^2 >0$, one has

30 $$\begin{aligned} | {\dot{\omega }}_{RN}| < 1.86 \times 10^{-3} \mathrm{rad}/\mathrm{year} \approx 11.625~ {\dot{\omega }}_{GR}, \end{aligned}$$

therefore,

31 $$\begin{aligned} 0< |Q| < 8.3516 M, \end{aligned}$$

with $95 \%$ C. L. As it was noted in [1] in 2018 after the pericenter passage of S2 star the current uncertainties of $| {\dot{\omega }}|$ will be improved by a factor 2, so for a tidal charge with $Q^2 < 0$, one has

32 $$\begin{aligned}&{\dot{\omega }}_{RN} < 6.9 \times 10^{-4} \mathrm{rad}/\mathrm{year} \approx 4.31~ {\dot{\omega }}_{GR}, \end{aligned}$$

33 $$\begin{aligned}&\quad -25.875 M^2< Q^2 <0, \end{aligned}$$

For $Q^2 >0$, one has

34 $$\begin{aligned} | {\dot{\omega }}_{RN}| < 9.1 \times 10^{-4} \mathrm{rad}/\mathrm{year} \approx 5.69~ {\dot{\omega }}_{GR}, \end{aligned}$$

therefore,

35 $$\begin{aligned} 0< |Q| < 5.80 M, \end{aligned}$$

One could expect that subsequent observations with VLT, Keck, GRAVITY, E-ELT and TMT will significantly improve an observational constraint on $| {\dot{\omega }}|$, therefore, one could expect that a range of possible values of *Q* parameter would be essentially reduced.

As it was noted in paper [1], currently Keck astrometric uncertainty is around $\sigma = 0.16~\hbox {mas}$, therefore, an angle $\delta =2 \sigma$ (or two standard deviations) is measurable with around 95% C.L. In this case $\Delta \theta (GR)_{S2}= 2.59 \delta$ for S2 star where we adopt $\Delta \theta (GR)_{S2} \approx 0.83$. Assuming that GR predictions about orbital precession will be confirmed in the next 16 years with $\delta$ accuracy (or $\left| \dfrac{\pi Q^2}{ML}\right| \lesssim \delta$), one could constrain *Q* parameter

36 $$\begin{aligned} |Q^2| \lesssim 2.32 M^2, \end{aligned}$$

where we wrote absolute value of $Q^2$ since for a tidal charge $Q^2$ could be negative. For negative $Q^2$ this estimate is better than estimate considered in [6] ($Q^2 \approx - 6.4 M^2$), however, the estimate (36) is slightly more worse than $Q^2 \approx 1.6 M^2$.

If we adopt uncertainty $\sigma _{TMT} = 0.015~\hbox {mas}$ for TMT-like scenario as it was used in [1] ($\delta _{TMT}=2\sigma _{TMT}$) or in this case $\Delta \theta (GR)_{S2}= 27.67 \delta _{TMT}$ for S2 star and assuming again that GR predictions about orbital precession of S2 star will be confirmed with $\delta _{TMT}$ accuracy (or $\left| \dfrac{\pi Q^2}{ML}\right| \lesssim \delta _{TMT}$) , one could conclude that

37 $$\begin{aligned} |Q^2| \lesssim 0.216 M^2, \end{aligned}$$

or based on results of future observations one could expect to reduce significantly a possible range of $Q^2$ parameter in comparison with a possible hypothetical range of $Q^2$ parameter which was discussed in [5, 6].

Recently the GRAVITY team reported about a discovery of post-Newtonian gravitational redshift near S2 star pericenter passage [68]. Assuming $f=0$ corresponds to the Newtonian case and $f=1$ corresponds to the first post-Newtonian correction of GR, the GRAVITY collaboration estimated *f*-value from observational data comparing precessions for Schwarzschild and Newtonian approaches and they concluded that the *f*-value must be much closer to GR value or more precisely $f=0.94 \pm 0.09$ [68] (see also discussions in [69]). If we adopt uncertainty $\sigma _\mathrm{GRAVITY} = 0.030~\hbox {mas}$ of the GRAVITY facilities [68] and assuming again that GR predictions on orbital precession of S2 star will be confirmed with $\delta _\mathrm{GRAVITY}=2\sigma _\mathrm{GRAVITY}$ accuracy (or $\left| \dfrac{\pi Q^2}{ML}\right| \lesssim \delta _\mathrm{GRAVITY}$), one could conclude that

38 $$\begin{aligned} |Q^2| \lesssim 0.432 M^2, \end{aligned}$$

or based on results of future GRAVITY observations one could expect to reduce significantly a possible range of $Q^2$ parameter in comparison with a possible range of $Q^2$ parameter constrained with current and future Keck data.

### Estimates of $\Lambda$-term constraints

In this subsection we assume that $Q=0$. One could re-write orbital precession in dimensional form

39 $$\begin{aligned} {\dot{\omega }}_{\Lambda } = \frac{\pi \Lambda c^2 a^3 \sqrt{1-e^2}}{PGM}. \end{aligned}$$

Dependences of functions ${\dot{\omega _{\Lambda }}}$ and ${\dot{\omega _{GR}}}$ on eccentricity and semi-major axis are different and orbits with higher semi-major axis and smaller eccentricity could provide a better estimate of $\Lambda$-term (the S2 star orbit has a rather high eccentricity). However, we use observational constraints for *S*2 star. For positive $\Lambda$, one has relativistic advance and

40 $$\begin{aligned} {\dot{\omega _{\Lambda }}} < 1.54 \times 10^{-3} \mathrm{rad}/\mathrm{yr} \approx 9.625~ {\dot{\omega _{GR}}}, \end{aligned}$$

or

41 $$\begin{aligned} 0< {\Lambda } < 3.9 \times 10^{-39} \mathrm{cm}^{-2}, \end{aligned}$$

for $\Lambda <0$ one has

42 $$\begin{aligned} 0< -\Lambda < 4.68 \times 10^{-39} \mathrm{cm}^{-2}, \end{aligned}$$

if we use current accuracy of Keck astrometric measurements $\sigma = 0.16~\hbox {mas}$ and monitor S2 star for 16 years and assume that additional apocenter shift ($2\sigma$)could be caused by a presence of $\Lambda$-term, one obtains

43 $$\begin{aligned} |{\Lambda }| < 1.56 \times 10^{-40} \mathrm{cm}^{-2}, \end{aligned}$$

while for TMT-like accuracy $\delta _{TMT} = 0.015~\hbox {mas}$ one has

44 $$\begin{aligned} |{\Lambda }| < 1.46 \times 10^{-41} \mathrm{cm}^{-2}. \end{aligned}$$

As one can see, constraints on cosmological constant from orbital precession of bright stars near the Galactic Center are much weaker than not only its cosmological estimates but also than its estimates from Solar system data [64].

## Conclusions

We consider the first relativistic corrections for apocenter shifts in post-Newtonian approximation for the case of Reissner – Nordström–de-Sitter metric. Among different theoretical models have been proposed for the Galactic Center different black hole models are rather natural. Perhaps, assumptions about a presence of electric charge in the metric do not look very realistic because a space media is usually quasi-neutral, but the charged black holes are discussed in the literature see, for instance [70] and references therein. Moreover, a Reissner–Nordström metric could arise in a natural way in alternative theories of gravity like Reissner–Nordström solutions with a tidal charge in Randall–Sundrum model [2] (such an approach is widely discussed in the literature). Recently, it was found that Reissner–Nordström metric is a rather natural solution in Horndeski gravity [71] and in this case $Q^2$ parameter reflects an interaction with a scalar field and it could be also negative similarly to a tidal charge. In paper [71] it was expressed an opinion that the hairy black hole solutions look rather realistic and these objects could exist in centers of galaxies and if such objects (hairy black holes in Horndeski gravity) exist in nature, in particular in the Galactic Center, current and future advanced facilities such as GRAVITY [72], E-ELT [73], TMT [74] etc. may be very useful to detect signatures of black hole hairs of an additional dimension. Therefore, non-vanishing (positive or negative) $Q^2$ parameter is arisen due to a presence of extra dimension or in Horndeski gravity for black holes with a scalar hair. We outline a procedure to constrain $Q^2$ parameter with current and future observations of bright stars at the Galactic Center. Even current Keck facilities could constrain $Q^2$ better ($Q^2 \approx -2.32 M^2$) than with analysis of hypothetical variations of S2 brightness as it was suggested in [6].

Certainly, $\Lambda$-term should be present in the model, however, if we adopt its cosmological value it should be very tiny to cause a significant impact on relativistic precession for trajectories of bright stars. If we have a dark energy instead of cosmological constant, one should propose ways to evaluate dark energy for different cases, therefore, one could constrain $\Lambda$-term from observations as it was noted in [47] analyzing impact of $\Lambda$-term on observational phenomena near the Galactic Center (similarly to the cases where an impact of $\Lambda$-term has been analyzed for effects in Solar system [64, 75, 76]).
